# Progress Toward Measles Preelimination — African Region, 2011–2012

**Published:** 2014-04-04

**Authors:** Balcha G. Masresha, Reinhard Kaiser, Messeret Eshetu, Reggis Katsande, Richard Luce, Amadou Fall, Annick R.G.A. Dosseh, Boubker Naouri, Charles R. Byabamazima, Robert Perry, Alya J. Dabbagh, Peter Strebel, Katrina Kretsinger, James L. Goodson, Deo Nshimirimana

**Affiliations:** 1 Immunization and Vaccine Development Program, World Health Organization (WHO) Regional Office for Africa, Brazzaville, Congo; 2 Expanded Program on Immunization, WHO Regional Office for Africa, Inter-Country Support Team, Harare, Zimbabwe; 3 Expanded Program on Immunization, WHO Regional Office for Africa, Inter-Country Support Team, Libreville, Gabon; 4 Expanded Program on Immunization, WHO Regional Office for Africa, Inter-Country Support Team, Ouagadougou, Burkina Faso; 5 Global Immunization Division, Center for Global Health, CDC; 6 Department of Immunization, Vaccines, and Biologicals, WHO, Geneva, Switzerland

In 2008, the 46 member states of the World Health Organization (WHO) African Region (AFR) adopted a measles preelimination goal to reach by the end of 2012 with the following targets: 1) >98% reduction in estimated regional measles mortality compared with 2000, 2) annual measles incidence of fewer than five reported cases per million population nationally, 3) >90% national first dose of measles-containing vaccine (MCV1) coverage and >80% MCV1 coverage in all districts, and 4) >95% MCV coverage in all districts by supplementary immunization activities (SIAs) (1). Surveillance performance objectives were to report two or more cases of nonmeasles febrile rash illness per 100,000 population, one or more suspected measles cases investigated with blood specimens in ≥80% of districts, and 100% completeness of surveillance reporting from all districts (1). This report updates previous reports (2–4) and describes progress toward the measles preelimination goal during 2011–2012. In 2012, 13 (28%) member states had >90% MCV1 coverage, and three (7%) reported >90% MCV1 coverage nationally and >80% coverage in all districts. During 2011–2012, four (15%) of 27 SIAs with available information met the target of >95% coverage in all districts. In 2012, 16 of 43 (37%) member states met the incidence target of fewer than five cases per million, and 19 of 43 (44%) met both surveillance performance targets. In 2011, the WHO Regional Committee for AFR established a goal to achieve measles elimination* by 2020. To achieve this goal, intensified efforts to identify and close population immunity gaps and improve surveillance quality are needed, as well as committed leadership and ownership of the measles elimination activities and mobilization of adequate resources to complement funding from global partners.

## Immunization Activities

WHO and the United Nations Children’s Fund (UNICEF) use data from administrative records and surveys reported annually by member states through the Joint Reporting Form (JRF)† to estimate MCV1 coverage among children aged 1 year. Since 2003, member states also have reported the proportion of districts reaching ≥80% MCV1 coverage. Estimates of MCV1 coverage in AFR were 74% in 2011 and 73% in 2012 (Table 1). The number of member states with >90% MCV1 coverage was 14 (30%) in 2011 and 13 (28%) in 2012 (Table 1). MCV1 coverage was >90% nationally and >80% in all districts in four (9%) of 44 member states reporting district coverage data in 2011 and three (7%) of 44 in 2012. By the end of 2012, 12 (26%) member states had introduced a second dose of measles containing-vaccine (MCV2) into the routine vaccination schedule.

During 2011–2012, approximately 133 million children were vaccinated during 35 measles SIAs (Table 2). Of these SIAs, 23 (66%) had >95% national level administrative coverage, and of the 27 with available information, four (15%) had >95% MCV administrative coverage in all districts. Among the 20 SIAs that had a post-SIA coverage survey, 19 (95%) had lower coverage estimated by survey than by administrative report (Table 2). At least one other child health intervention was delivered in 23 (66%) SIAs (Table 2).

## Surveillance Activities

In 2012, the WHO Global Measles and Rubella Laboratory Network§ supported standardized methods and quality assurance measures in 44 laboratories in 42 member states. Measles case-based surveillance includes individual case investigation and blood specimen collection for laboratory testing (5). Suspected measles cases are confirmed on the basis of laboratory findings, an epidemiologic link, or clinical criteria.¶ During outbreaks, nasopharyngeal swab specimens are collected to identify measles virus genotypes.

During 2011–2012, 43 (93%) member states reported measles case-based surveillance data, and all member states reported annually through the JRF the number of measles cases. In 2012, 19 (44%) member states met both targets of two or more cases of nonmeasles febrile rash illness per 100,000 population and one or more suspected measles cases investigated with blood specimens in ≥80% of districts, 14 (33%) met one of the targets but did not meet the other target, and 10 (23%) did not meet either of the targets (Figure).

## Measles Incidence and Measles Virus Genotypes

On the basis of measles case-based surveillance data, the number of confirmed measles cases decreased from 43,800 in 2011 to 25,905 in 2012, and confirmed measles incidence per million population decreased from 50.4 to 29.0 (Table 1). In 2012, 16 of 43 (37%) member states met the incidence target of fewer than five cases per million. The number of measles cases reported through the JRF was 194,364 in 2011 and 106,052 in 2012. Measles incidence per million population was 223.6 in 2011 and 118.8 in 2012 (Table 1). During 2011–2012, measles virus genotype results were reported from 20 (43%) member states; the predominant genotypes detected were B3 in all 20 reporting member states; B2 in Angola, the Democratic Republic of the Congo (DRC), and Namibia; and D4 in Uganda.**

What is already known on this topic?

During 2001–2008, measles cases reported through the World Health Organization–United Nations Children’s Fund (WHO-UNICEF) Joint Reporting Forms (JRF) decreased in the African Region (AFR) by 92%, from 492,116 to 37,012; however, during 2009–2010, the region was affected by major measles outbreaks, and the number of officially reported cases increased to 199,174 in 2010.

What is added by this report?

The numbers of JRF-reported measles cases in AFR were 194,364 in 2011 and 106,052 in 2012. By the end of 2012, the first dose of measles vaccine coverage in AFR was 73% (WHO-UNICEF estimate), 13 (28%) member states reported >90% first dose of measles vaccine coverage, and 16 (37%) member states had met the incidence target of fewer than five cases per million. Of 35 measles supplementary immunization activities (SIAs) conducted during 2011–2012, 23 (66%) reported administrative coverage rates >95%. Despite this progress, the region fell short of the 2012 measles preelimination goal.

What are the implications for public health practice?

To achieve the measles elimination target in AFR by 2020, efforts must be intensified at the global and national levels to implement strategies that include 1) closing gaps in population immunity through adopting and implementing updated policy recommendations to decrease missed opportunities, including routine immunization of unvaccinated older children, 2) sustaining implementation of the “reaching every district” approach to increase the coverage and quality of routine immunization services, 3) conducting high-quality SIAs, and 4) using SIAs to improve routine immunization services.

### Discussion

Despite substantial progress and an 88% reduction in estimated measles mortality in AFR (from 354,900 to 41,400) during 2000–2012 (6), the measles 2012 preelimination goal was not reached. Major outbreaks occurred during 2009–2010, and reported measles cases have remained above the historic low of 37,012 cases in 2008 (2,3). During 2011–2012, large outbreaks occurred in a small number of member states; 89% of cases in 2011 were from four member states (Chad, DRC, Nigeria, and Zambia), and 88% of cases in 2012 were from five member states (Angola, Burkina Faso, DRC, Ethiopia, and Nigeria). Various outbreak investigation activities conducted in these outbreaks indicated that the primary causes were an accumulation of susceptible older children and adolescents, shifting susceptibility towards older age groups, and continued gaps in reaching all children with 2 doses of measles vaccine at national and subnational levels through routine vaccination or periodic follow-up SIAs.

Annual measles cases in AFR reported through the JRF have been consistently higher than those reported through case-based surveillance. According to WHO guidelines, the total number of confirmed cases reported to the measles case-based surveillance system should match the total number of measles cases reported through the JRF. In 2012, 13 member states reported considerably more cases through the JRF than case-based surveillance.†† These differences might be attributable to classification errors, reporting errors, difficulties in capturing large outbreaks through the case-based system, or reliance on aggregate summary reporting of notifiable diseases through the Integrated Disease Surveillance and Response system.§§ Limited implementation of case-based surveillance in some health facilities, incomplete preparation and reporting of line lists during outbreaks, and insufficient personnel to enter all surveillance data into databases might contribute to underreporting through measles case-based surveillance.

The proportion of member states meeting both case-based surveillance performance indicators increased from 35% in 2009 (3) to 44% in 2012. Measles surveillance systems in member states not attaining objectives for surveillance indicators might lack the sensitivity to allow rapid detection and response to outbreaks. Monitoring district-level surveillance performance indicators can help member states to identify and prioritize support for areas needing to improve performance; conducting adequate outbreak investigations could rapidly identify and characterize outbreaks and guide response activities.

The findings in this report are subject to at least three limitations. First, MCV coverage estimates likely include errors from inaccurate estimates of the size of target populations, inaccurate reporting of doses delivered, and inclusion of SIA doses given to children outside the target age group. Second, surveillance data underestimate the actual number of cases because not all patients with measles seek care and not all of those who seek care are reported. Finally, some member states maintain multiple reporting systems for measles and might, like DRC, report in the JRF aggregate, unconfirmed cases rather than confirmed cases generated from case-based surveillance.

The Global Vaccine Action Plan and the Measles and Rubella Initiative¶¶ Strategic Plan provide key strategies and targets for measles elimination in five regions by 2020 (7,8). In September 2011, the WHO Regional Committee for AFR established a goal of measles elimination by 2020 (9). The regional strategic plan for measles elimination (2012–2020) outlines the key programmatic focus, and the approaches to follow to achieve measles elimination. In AFR member states, intensified efforts to increase coverage with 2 doses of MCV include implementing updated policies to decrease missed opportunities, including opening multidose vials even when few eligible children are present, immunizing unvaccinated children aged ≤5 years through routine immunization services, sustaining the implementation of the “reaching every district” approach (10), using SIAs to improve routine immunization services, and introducing a second dose in the routine immunization schedule once criteria are met.*** To ensure high population immunity, member states should also conduct high-quality, well-monitored SIAs that are routinely evaluated through coverage surveys. SIA target age groups should be based on national measles epidemiology determined by surveillance and immunization data.

Member states are encouraged to mobilize adequate additional resources to complement the funding from global partners to achieve their goal of measles elimination. In addition to funding from the Measles and Rubella Initiative and other organizations, the GAVI Alliance is providing funding to support the introduction of a second dose of measles vaccine in routine immunization; measles SIAs in Chad, DRC, Ethiopia, and Nigeria; and the introduction of rubella vaccine through wide–age range measles-rubella vaccination campaigns.

## Figures and Tables

**FIGURE:**
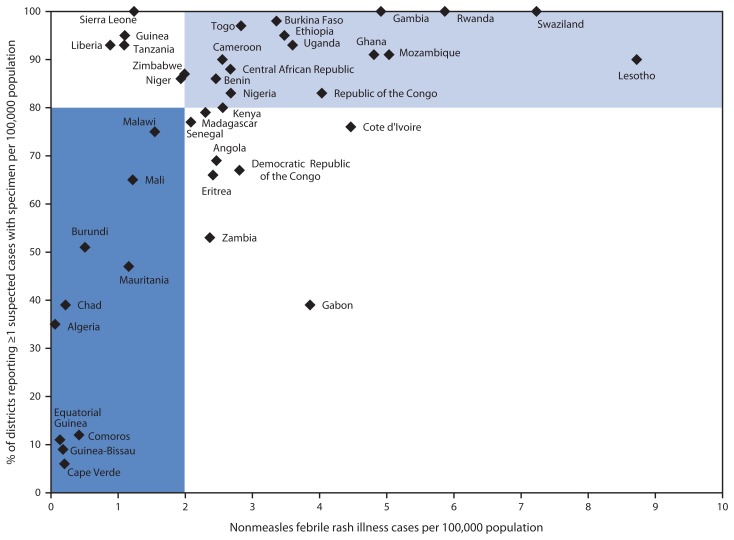
Measles surveillance performance, by member state* — World Health Organization African Region, 2012 * In the light blue area, member states met both targets of two or more cases of nonmeasles febrile rash illness per 100,000 population and one or more suspected measles cases investigated with blood specimens in ≥80% of districts. In white areas, member states met at least one target. In the dark blue area, member states did not meet any of the two targets. Not shown: Botswana (percentage of districts reporting one or more suspected measles cases with specimen per 100,000 population = 96; nonmeasles febrile rash illness rate per 100,000 population = 15.7), Namibia (94 and 15.5, respectively), and South Africa (100 and 12.7, respectively).

**TABLE 1:** Reported coverage with the first dose of measles-containing vaccine (MCV1), number of confirmed measles cases, confirmed measles incidence, and proportion of measles cases in children aged <5 years, by member state — World Health Organization (WHO) African Region, 2011 and 2012

	2011						2012					
		
Member state	% coverage with MCV1 (WHO-UNICEF estimate)*	No. of confirmed† measles cases (case-based surveillance)	Measles incidence per million population (case-based surveillance)	No. of measles cases (JRF)*	Measles incidence per million population (JRF)*	Proportion of measles cases in children aged <5 yrs (%) (case-based surveillance)§	% coverage with MCV1 (WHO-UNICEF estimate)*	No. of confirmed† measles cases (case-based surveillance)	Measles incidence per million population (case-based surveillance)	No. of measles cases (JRF)*	Measles incidence per million population (JRF)*	Proportion of measles cases in children aged <5 yrs (%) (case-based surveillance)§
Algeria	95	126	3.3	112	3.0	27.8	95	6	0.2	18	0.5	NA
Angola	88	190	9.4	1,449	71.8	65.3	97	4,416	212.1	4,458	214.1	70.8
Benin	72	431	44.1	426	43.6	69.1	72	286	28.5	288	28.7	62.2
Botswana	94	7	3.5	8	4.0	NA	94	10	5.0	7	3.5	NA
Burkina Faso	89	285	17.8	860	53.8	47.4	87	815	49.5	7,362	447.3	35.8
Burundi	93	65	6.8	129	13.5	76.9	93	49	5.0	49	5.0	83.7
Cameroon	76	914	43.2	504	23.8	66.1	82	630	29.0	609	28.1	71.4
Cape Verde	96	0	0.0	0	0.0	NA	96	0	0.0	0	0.0	NA
Central African Republic	49	679	153.1	679	153.1	59.3	49	68	15.0	141	31.2	58.8
Chad	54	146	12.1	8,650	716.1	41.4	64	140	11.2	120	9.6	48.2
Comoros	87	3	4.3	3	4.3	NA	85	0	0.0	1	1.4	NA
Cote d’Ivoire	49	631	32.5	628	32.4	70.4	85	153	7.7	137	6.9	50.5
DRC	74	1,519	23.8	133,802	2,092.9	75.0	73	2,353	35.8	72,029	1,096.2	68.5
Equatorial Guinea	51	0	0.0	0	0.0	NA	51	8	10.9	1,190	1,616.8	NA
Eritrea	99	14	2.4	48	8.1	7.1	99	95	15.5	194	31.6	7.4
Ethiopia	68	3,556	39.8	3,255	36.4	30.4	66	4,514	49.2	4,347	47.4	40.6
Gabon	72	2	1.3	2	1.3	NA	71	5	3.1	2	1.2	NA
Gambia	91	0	0.0	0	0.0	NA	95	0	0.0	0	0.0	NA
Ghana	91	137	5.5	120	4.8	28.2	88	354	14.0	1,613	63.6	50.0
Guinea	58	7	0.6	11	1.0	NA	58	7	0.6	6	0.5	NA
Guinea-Bissau	69	0	0.0	0	0.0	NA	69	5	3.0	0	0.0	NA
Kenya	87	2,461	58.6	2,395	57.0	41.1	93	2,380	55.1	NR	NR	44.6
Lesotho	85	0	0.0	172	84.7	NA	85	0	0.0	179	87.2	NA
Liberia	71	24	5.9	279	68.4	41.7	80	4	1.0	43	10.3	NA
Madagascar	70	1	0.0	0	0.0	NA	69	3	0.1	2	0.1	NA
Malawi	96	21	1.4	26	1.7	42.9	90	10	0.6	11	0.7	NA
Mali	56	25	1.7	24	1.7	40.0	59	365	24.6	341	23.0	45.0
Mauritania	67	188	50.8	234	63.2	24.5	75	4	1.1	35	9.2	NA
Mauritius	99	NR	NR	2	1.6	NR	99	NR	NR	0	0.0	NR
Mozambique	82	155	6.3	177	7.2	50.0	82	135	5.4	145	5.8	49.6
Namibia	74	86	38.8	79	35.6	40.7	76	97	42.9	86	38.1	58.1
Niger	76	775	46.9	771	46.7	35.7	73	311	18.1	272	15.9	47.9
Nigeria	57	15,970	97.3	18,843	114.8	74.6	42	5,938	35.2	6,447	38.2	48.6
Republic of the Congo	90	142	33.6	315	74.6	62.0	80	257	59.3	260	59.9	66.8
Rwanda	95	28	2.5	31	2.8	57.1	97	79	6.9	75	6.5	29.1
Sao Tome and Principe	91	NR	NR	0	0.0	NR	92	NR	NR	0	0.0	NR
Senegal	84	22	1.7	18	1.4	40.9	84	54	3.9	46	3.4	57.1
Seychelles	99	NR	NR	0	0.0	NR	98	NR	NR	0	0.0	NR
Sierra Leone	80	16	2.7	1,865	318.0	62.5	80	41	6.9	678	113.4	56.1
South Africa	78	155	3.0	92	1.8	69.0	79	38	0.7	32	0.6	63.2
Swaziland	98	0	0.0	0	0.0	NA	88	0	0.0	0	0.0	NA
Togo	72	168	26.0	187	28.9	59.5	72	263	39.6	238	35.8	52.1
Uganda	75	126	3.6	3,312	94.2	80.2	82	723	19.9	2,027	55.8	71.5
Tanzania	93	1,570	33.9	1,622	35.0	65.6	97	738	15.4	1,668	34.9	49.9
Zambia	83	13,153	964.7	13,234	970.7	48.0	83	558	39.6	896	63.7	57.7
Zimbabwe	90	2	0.1	0	0.0	NA	90	0	0.0	0	0.0	NA
**Regional total**	**74**	**43,800**	**50.4**	**194,364**	**223.6**	**58.3**	**73**	**25,905**	**29.0**	**106,052**	**118.8**	**53.9**

**Abbreviations:** UNICEF = United Nations Children’s Fund; JRF = Joint Reporting Form; NA = not applicable; DRC = Democratic Republic of the Congo; NR = not reported.

* Data available at http://www.who.int/immunization_monitoring/data/data_subject/en/index.html.

† Confirmed cases were defined by laboratory criteria, epidemiologic linkage, and/or clinical criteria: laboratory-confirmed was defined as having measles-specific immunoglobulin M-positive test result and not receiving a measles vaccination during the 30 days before rash onset; epidemiologically linked was defined as meeting the suspected measles case definition and having contact (i.e., lived in the same district or an adjacent district, with plausibility of transmission) with a patient with a laboratory-confirmed measles case with rash onset within the preceding 30 days; clinically compatible was defined as meeting the case definition of measles, with no sample available for laboratory testing and no evidence of epidemiologic linkage to a laboratory-confirmed case. A suspected measles case was defined as an illness characterized by rash, fever, and one or more of the following symptoms: conjunctivitis, coryza, and cough, or any patient in whom the clinician suspected measles.

§ Countries with ≥10 cases with available age information.

**TABLE 2:** Characteristics of measles supplementary immunization activities (SIAs),*† by year and member state— World Health Organization African Region, 2011 and 2012

Year	Member state§	Age group targeted (mos)	Children reached (administrative coverage) in targeted age group		Proportion of districts with ≥95% coverage (%)	Post-SIA coverage survey (%)	Other interventions

			No.	(%)			
2011	Angola	9–59	4,635,248	(85)	(17)		OPV, vitamin A, anthelminthics
	Benin	9–59	1,411,065	(104)	(93)	(83)	
	Burkina Faso	9–59	2,865,517	(113)	(100)		
	Central African Republic	9–47	515,452	(84)	(33)		OPV, vitamin A, anthelminthics
	Cote d’Ivoire	9–59	5,820,653	(95)	(72)	(91)	OPV
	DRC						
		6–59	7,368,047	(98)			
		6–179	9,280,981	(100)			
	Equatorial Guinea	9–47	11,658	(50)			
	Ethiopia				(91)	(88)	OPV, vitamin A, anthelminthics
	Rollover campaigns¶	9–47	757,421	(98)			
	Outbreak response immunization	6–179	7,034,264	(96)			
	Gambia	9–59	307,613	(95)	(31)	(93)	Vitamin A
	Liberia	6–59	572,981	(103)	(60)	(99)	OPV, vitamin A, anthelminthics
	Mali	9–59	4,616,957	(94)	(62)		
	Mauritania	9–59	510,155	(96)		(90)	
	Mozambique	6–59	3,985,564	(104)	(86)	(81)	OPV, vitamin A, anthelminthics
	Nigeria	9–59	28,435,589	(100)	(52)	(94)	OPV, vitamin A, anthelminthics
	Tanzania	9–59	6,686,663	(97)	(60)	(92)	OPV and tetanus toxoid vaccine, vitamin A, anthelminthics
2012	Burundi	6–59	1,459,304	(102)	(82)		Vitamin A, anthelminthics
	Cameroon	9–59	3,562,478	(102)	(78)	(78)	OPV, vitamin A, anthelminthics
	Chad	9–59	2,270,772	(111)	(83)		OPV
	DRC						
		6–59	2,972,570	(104)			
		6–179	3,605,069	(101)			
	Equatorial Guinea	9–59	49,578	(58)			
	Eritrea	9–47	277,928	(74)	(16)	(96)	Vitamin A
	Gabon	6–59	169,999	(67)	(20)		Vitamin A, anthelminthics
	Guinea	9–59	2,275,245	(103)	(92)	(91)	OPV
	Guinea-Bissau	9–59	220,826	(89)	(18)	(68)	Vitamin A, anthelminthics
	Kenya	9–59	5,554,153	(92)	(64)	(88)	OPV, vitamin A
	Namibia	9–179	885,259	(91)	(100)	(89)	OPV and tetanus toxoid vaccine, vitamin A, anthelminthics
	Niger	9–179	7,780,724	(100)	(93)	(97)	Anthelminthics
	Sao Tome and Principe	9–59	22,476	(105)	(100)		
	Sierra Leone	9–59	1,179,605	(102)	(100)	(96)	Vitamin A, anthelminthics
	Uganda	6–59	6,283,441	(100)	(73)	(95)	OPV, vitamin A, anthelminthics
	Zambia	9–179	7,503,515	(116)	(93)	(96)	OPV, vitamin A, anthelminthics
	Zimbabwe	6–59	1,613,437	(103)	(84)	(95)	OPV, vitamin A

**Abbreviations:** OPV = oral poliovirus vaccine; DRC = Democratic Republic of the Congo.

* Data available at http://www.who.int/immunization/monitoring_surveillance/data/subject.

† SIAs generally are carried out using two approaches. An initial, nationwide catch-up SIA targets all children aged 9 months-14 years; it has the goal of eliminating susceptibility to measles in the general population. Periodic follow-up SIAs then target all children born since the last SIA. Follow-up SIAs generally are conducted nationwide every 2–4 years and generally target children aged 9–59 months; their goal is to eliminate any measles susceptibility that has developed in recent birth cohorts and to protect children who did not respond to the first measles vaccination. The exact age range for follow-up SIAs depends on the age-specific incidence of measles, coverage with measles-containing vaccine through routine services, and the time since the last SIA.

§ Type of SIA is national if not indicated otherwise.

¶ Rollover campaigns were conducted in phases and spread out during >1 calendar year.

## Footnotes

* Measles elimination is defined as the absence of endemic measles virus transmission in a defined geographic area (e.g., region or country) for ≥12 months in the presence of a well-performing surveillance system.

† WHO and UNICEF jointly collect information through a standard questionnaire, the JRF, sent to all member states. Information collected in the JRF includes estimates of national immunization coverage, reported cases of vaccine-preventable diseases, immunization schedules, and indicators of immunization system performances. Additional information available at http://www.who.int/immunization/monitoring_surveillance/routine/reporting/reporting/en. JRF data are available at http://www.who.int/immunization_monitoring/data/data_subject/en/index.html.

§ This network includes 44 national laboratories; three are also regional reference laboratories (in Abidjan, Cote d’Ivoire; Entebbe, Uganda; and Johannesburg, South Africa). Member states currently not participating in case-based surveillance and without national laboratories are Mauritius, Sao Tome and Principe, and Seychelles.

¶ Cases that meet the WHO clinical case definition of measles for which no adequate specimen was collected and cannot be epidemiologically linked to a laboratory-confirmed case of measles.

** Measles genotypes contributed by WHO Global Measles and Rubella Laboratory Network (Measles Nucleotide Surveillance Database, available at http://www.who-measles.org/Public/Web_Front/main.php ); National Institute for Communicable Diseases, Johannesburg, South Africa (Sheilagh Smit); Institute Pasteur, Abidjan, Cote d’Ivoire (Herve Kadjo); National Laboratory Democratic Republic of the Congo (Elisabeth Simbu Pukuta); CDC Measles Team, Atlanta, Georgia, United States (Raydel Anderson and Paul Rota); Uganda Virus Institute, Entebbe, Uganda (Barnabas Bakamutumaho, Prossy Namuwulya).

†† Burkina Faso, Central African Republic, DRC, Equatorial Guinea, Eritrea, Ghana, Lesotho, Liberia, Mauritania, Sierra Leone, Uganda, Tanzania, and Zambia.

§§ The Integrated Disease Surveillance and Response Strategy was adopted in resolution AFR/RC48/R2 in September 1998 at the 48th session of the WHO Regional Committee for Africa as a strategy for improving the availability and use of data for public health action at all levels and for implementing comprehensive public health surveillance and response systems in countries in the region.

¶¶ The Measles and Rubella Initiative is a partnership led by the American Red Cross, the United Nations Foundation, CDC, UNICEF, and WHO. Other member partners include the Bill and Melinda Gates Foundation; the Canadian International Development Agency; the Church of Jesus Christ of Latter-Day Saints, the GAVI Alliance, the International Federation of Red Cross and Red Crescent Societies, the United Kingdom Department for International Development, other foundations and organizations, and governments of countries affected by measles.

*** The 2008 AFR Measles Technical Advisory Group set criteria to consider the introduction MCV2: achievement of MCV1 coverage >80%, maintained for at least 3 consecutive years using WHO/UNICEF best estimates of vaccination coverage, and attainment of one of the two primary measles surveillance performance indicators for ≥2 consecutive years. The two indicators are 1) a nonmeasles febrile rash illness rate of two or more cases per 100,000 population per year (i.e., suspected measles cases investigated and discarded as nonmeasles cases), and 2) at least one suspected measles case investigated with blood specimens in >80% of districts per year. After successful introduction of MCV2, the implementation of periodic follow-up measles SIAs should continue until national MCV1 and MCV2 coverage reach and are sustained at ≥90% and the two primary measles surveillance performance indicators are met and sustained for ≥2 years.
